# Rapamycin-Induced Autophagy Promotes the Chondrogenic Differentiation of Synovium-Derived Mesenchymal Stem Cells in the Temporomandibular Joint in Response to IL-1*β*

**DOI:** 10.1155/2020/4035306

**Published:** 2020-10-22

**Authors:** Wenjing Liu, Haiyun Luo, Ruolan Wang, Yiyuan Kang, Wenting Liao, Yangpeng Sun, Guodong Chen, Longquan Shao

**Affiliations:** ^1^Shunde Hospital, Southern Medical University (The First People's Hospital of Shunde), Foshan 528308, China; ^2^Stomatological Hospital, Southern Medical University, Guangzhou 510280, China; ^3^Nanfang Hospital, Southern Medical University, Guangzhou 510515, China; ^4^Department of Oral and Maxillofacial Surgery, Guanghua School of Stomatology, Hospital of Stomatology, Sun Yat-sen University, Guangzhou 510055, China

## Abstract

Cartilage defects in temporomandibular disorders (TMD) lead to chronic pain and seldom heal. Synovium-derived mesenchymal stem cells (SMSCs) exhibit superior chondrogenesis and have become promising seed cells for cartilage tissue engineering. However, local inflammatory conditions that affect the repair of articular cartilage by SMSCs present a challenge, and the specific mechanism through which the function remains unclear. Thus, it is important to explore the chondrogenesis of SMSCs under inflammatory conditions of TMD such that they can be used more effectively in clinical treatment. In this study, we obtained SMSCs from TMD patients with severe cartilage injuries. In response to stimulation with IL-1*β*, which is well known as one of the most prevalent cytokines in TMD, MMP13 expression increased, while that of SOX9, aggrecan, and collagen II decreased during chondrogenic differentiation. At the same time, IL-1*β* upregulated the expression of mTOR and decreased the ratio of LC3-II/LC3-I and the formation of autophagosomes. Further study revealed that rapamycin pretreatment promoted the migration of SMSCs and the expression of chondrogenesis-related markers in the presence of IL-1*β* by inducing autophagy. 3-Benzyl-5-((2-nitrophenoxy)methyl)-dihydrofuran-2(3H)-one (3BDO), a new activator of mTOR, inhibited autophagy and increased the expression of p-GSK3*β*ser9 and *β*-catenin, simulating the effect of IL-1*β* stimulation. Furthermore, rapamycin reduced the expression of mTOR, whereas the promotion of LC3-II/LC3-I was blocked by the GSK3*β* inhibitor TWS119. Taken together, these results indicate that rapamycin enhances the chondrogenesis of SMSCs by inducing autophagy, and GSK3*β* may be an important regulator in the process of rapamycin-induced autophagy. Thus, inducing autophagy may be a useful approach in the chondrogenic differentiation of SMSCs in the inflammatory microenvironment and may represent a novel TMD treatment.

## 1. Introduction

Temporomandibular disorder (TMD) is a common disease in the maxillofacial region. Irreversibly damaged cartilage in TMD leads to severe pain and mandibular movement disorders. Currently, the primary TMD treatments, including occlusal plates, drugs, physical or psychological treatments, joint irrigation, or surgical intervention, can alleviate symptoms but cannot repair damaged cartilage.

With the development of regenerative medicine, stem cell-based tissue engineering technology provides a new opportunity for cartilage reconstruction. However, mesenchymal stem cells (MSCs) from different sources exhibit different characteristics. For instance, in vitro assays have shown that synovium-derived MSCs (SMSCs) exhibit stronger proliferation abilities, slower cell senescence, and better chondrogenic abilities than MSCs from other sources in vitro, such as bone marrow MSCs and adipose MSCs [1]. Thus, SMSCs may be the best candidate for articular cartilage repair. With the progress and development of research, the survival, proliferation, and differentiation properties of MSCs have been shown to differ with respect to the local disease [2]. The results of our previous study also showed that inflammatory factors associated with TMD stimulate the inflammatory secretion from SMSCs, which may not be conducive to chondrocyte regeneration [3]. Therefore, investigating the differential capacity and mechanisms of SMSCs in the inflammatory microenvironment is necessary to provide new insights into the TMD treatment.

Autophagy is an important mechanism for maintaining the balance of the intracellular environment by degrading cell metabolites and restoring functional cellular proteins. At the same time, autophagy plays a regulatory role in stem cell proliferation and multidirectional differentiation. Studies have focused on the effects of autophagy with respect to chronic inflammation of the synovium[4, 5]. However, whether autophagy plays a role in the chondrogenesis of SMSCs under inflammatory conditions has not yet been investigated. Glycogen synthase kinase 3*β* (GSK3*β*) regulates the degradation of *β*-catenin and affects the activity of the Wnt/*β*-catenin pathway, which plays an important role in cartilage regeneration and differentiation [6, 7]. However, the relationship between GSK3*β* and autophagy remains uncertain. A previous study demonstrated that GSK3*β* stimulates the unc-51 like kinase 1 (ULK1) to elicit autophagy [8], and subsequent studies showed that GSK3 positively regulates the mammalian target of rapamycin complex 1 (mTORC1) to inhibit autophagic activity^[^9^]^. Therefore, the effect of GSK3*β* on autophagy during the chondrogenic differentiation of SMSCs in the presence of inflammatory cytokines remains elusive.

As an important inflammatory cytokine in the initiation and development of TMD, IL-1*β* was widely used to simulate the inflammatory microenvironment of TMD in vitro[10, 11]. In this study, we showed that IL-1*β* inhibited the autophagy and chondrogenic differentiation of SMSCs. Rapamycin, a target for mTOR and an autophagy activator, promoted the chondrogenesis of SMSCs in response to IL-1*β* stimulation. Moreover, GSK3*β* inhibition disrupted the rapamycin-mediated activation of autophagy during chondrogenic induction. These findings may provide a theoretical foundation for the use of rapamycin in cell-based therapies in cartilage regeneration and TMD.

## 2. Materials and Methods

### 2.1. SMSCs Were Cultured In Vitro

The synovium intima specimens were obtained in open temporomandibular joint surgery from 10 TMD patients with severe cartilage tissue injuries. The study was approved by the Institutional Ethics Board of the Stomatology Hospital, Sun Yat-sen University (ERC-2017-8). The specimens were isolated from 25 to 64 years old patients without systemic diseases and local application of medicines, with 8 females and 2 males. The sample providers signed informed consent before surgery.

The obtained synovial membranes were cut into pieces and digested with 4 mg/mL type I collagenase (Sigma-Aldrich, USA) for 2.5 hours at 37°C. After centrifugation, the precipitates were cultured in low glucose Dulbecco's modified Eagle medium (DMEM; Gibco, USA) with 10% fetal bovine serum (FBS; Gibco, USA) at 37°C. The cells from each sample were mixed and passaged at a ratio of 1 : 3, and cells from passages 4–8 were used for all subsequent experiments.

### 2.2. Surface Phenotype Identification of SMSCs by Flow Cytometry

The cells were harvested and incubated with different surface marker antibodies, including CD73 (1 : 11; Miltenyi Biotec, Germany), CD44, CD90, CD105, CD11b, HLA-DR, CD45, CD34 (1 : 20; BD Biosciences, USA), and isotype control (1 : 20; BD Biosciences, USA), then washed with PBS. The cell fluorescence was detected through FC500 Flow Cytometer (Beckman Coulter, USA) and analyzed with the CXP Software (Beckman Coulter, USA).

### 2.3. Multilineage Differentiation of SMSCs

#### 2.3.1. Osteogenic Differentiation

SMSCs were cultured in six-well plates with osteogenic induction medium, which composed of high glucose DMEM (Gibco, USA), 10 mM sodium *β*-glycerophosphate (Santa Cruz Biotechnology, USA), 10% FBS, 50 *μ*g/L ascorbic acid-2-phosphate (Wako, Japan), and 100 nM dexamethasone (MP Biomedicals, USA). The control group was cultured with complete culture medium. After induction for 2 weeks, the cells in the plates were fixed and stained with alizarin red solution (Cyagen, USA). The gene expression levels of alkaline phosphatase (ALP) and runt-related transcription factor 2 (RUNX2) were quantitated by reverse-transcription quantitative polymerase chain reaction (qRT-PCR).

#### 2.3.2. Adipogenic Differentiation

The SMSCs of the induction group were replaced with adipogenic induction medium consisted of high-glucose DMEM, 0.5 *μ*M isobutyl methylxanthine (Sigma-Aldrich, USA), 10% FBS, 1 *μ*M dexamethasone (MP Biomedicals, USA), 10 *μ*g/mL insulin (Telenbiotech, China), and 200 *μ*M indomethacin (Sigma-Aldrich, USA). After 2 weeks of incubation, the cells were fixed and stained with oil red O (Cyagen, USA). The gene expression of peroxisome proliferator-activated receptor gamma (PPARG) 2 and lipoprotein lipase (LPL) were determined by qRT-PCR.

#### 2.3.3. Chondrogenic Differentiation

SMSCs (3 × 10^5^) were harvested and placed in a 15 mL centrifuge tube with chondrogenic induction medium (Stem Chondro Diff Kit; Invitrogen, USA). After incubation for 2 weeks, the cartilage pellets were fixed and paraffin-embedded. The sample sections were stained with alcian blue staining reagent for 30 min to detect the glycosaminoglycan and collagen II (1 : 100; Cell Signaling Technology, USA); immunohistochemical staining was performed following the manufacturers' instructions. The gene expression of aggrecan (ACAN), matrix metallopeptidase 13 (MMP13) and sex-determining region Y-type high mobility group box gene 9 (SOX9) in the chondrogenic pellets were detected by qRT-PCR. The control group was cultured with a complete culture medium instead of chondrogenic induction medium.

### 2.4. Reagent Preparation

IL-1*β* (PeproTech, USA) was dissolved in double-distilled water. Rapamycin (Selleck, USA) and 3-benzyl-5-((2-nitrophenoxy) methyl)-dihydrofuran-2(3H)-one (3BDO) (Selleck, USA) were dissolved in dimethyl sulfoxide (DMSO; MP Biomedicals, USA). The cells cultured in the medium without rapamycin and 3BDO were also contained 0.1% DMSO.

### 2.5. Immunohistochemical Staining

The sections for cartilage pellets were dewaxed and rehydrated and then incubated with 3% H_2_O_2_. After antigen retrieval, blocked with 5% bovine serum albumin (BSA, Leagene, China). The sections were incubated with primary antibody (Collagen II, 1 : 100, Cell Signaling Technology, USA; SOX9, 1 : 50, Santa Cruz Biotechnology, USA) overnight at 4°C. After washed with phosphate-buffered saline (PBS; Gibco, USA), the slides were incubated in secondary antibody (Boster, China), streptavidin-biotin complex, and 3,3′-diaminobenzidine (DAB; Boster, China) follow one another. The stained sections onto microscope slides were evaluated by light microscopy (Leica, Germany).

### 2.6. Immunofluorescence Assay

SMSCs were seeded on glass coverslips in 24-well plates and treated as the experiment group processing. After another 24 hours, the chondrogenic induction cells were fixed and subsequently treated with 0.3% Triton X-100 (MP Biomedicals, USA). After washed with PBS, the cells were then incubated in 5% BSA. Then, the cells were incubated with microtubule-associated protein light chain 3 (LC3) rabbit monoclonal antibody (1 : 100; Abcam, USA) overnight at 4°C. The negative control group was treated with 1% BSA instead of the primary antibody. The FITC-conjugated goat antirabbit IgG (1 : 100; Proteintech, USA) was added for 1 hour and then incubated with DAPI staining solution (Leagene, China) for 15 min. The coverslip was dried and sealed with antifluorescence quenching agent (Beyotime, China). The cells were observed under laser confocal microscopy (FV10i, Olympus, Japan).

### 2.7. Transmission Electron Microscopy

The cells were fixed in 2.5% glutaraldehyde and embedded with epoxy resin (Sigma-Aldrich, USA). The sample sections were stained with lead citrate (Sigma-Aldrich, USA) and assessed under the electron microscope (JEM-2100F, Hitachi, Japan).

### 2.8. Cell Migration Assay

SMSCs were seeded on a 24-well plate. After 80% confluence of cells, the complete culture media was replaced by low glucose DMEM with 1% FBS for 24 hours. A scratch was taken by a sterile pipette tip (1 mL) in each well. The exfoliated cells were removed with PBS and then added reagents according to the experiment grouping. The initial images were taken under an inverted microscope (Leica, Germany). After 24 hours, the cells were fixed and stained with crystal violet solution. The images were taken by using a stereomicroscope (Leica, Germany).

### 2.9. QRT-PCR Assay

The total RNA of each sample was extracted according to the manufacturer's instructions. Reverse transcription obtained cDNA using the prime script RT Kit (Takara, Japan). Finally, the relative mRNA expression levels for the RUNX2, ALP, LPL, PPARG2, SOX9, ACAN, and MMP13 were determined by PCR with SYBR Premix Ex TM Taq II (Takara, Japan). The glyceraldehyde 3-phosphate dehydrogenase (GAPDH) expression was used as the reference for relative quantitative statistics. The primers for these genes are listed in Table 1.

### 2.10. Western Blot Analysis

The proteins were extracted and measured using the BCA Protein Assay Kit (Thermo Fisher, USA). The denatured proteins were electrophoretically separated and transferred onto polyvinylidene difluoride (PVDF) membranes (Millipore, USA). After blocked with 5% BSA for 1 hour at room temperature, the membranes were immersed with the primary antibodies (Collagen II, 1 : 1000, Cell Signaling Technology, USA; LC3, 1 : 1000, Abcam, USA; p-mTOR, 1 : 1000, Cell Signaling Technology, USA; mTOR, 1 : 1000, Cell Signaling Technology, USA; GSK3*β*, 1 : 1000, Huabio, China; p-GSK3*β*ser9, 1 : 1000, Huabio, China; *β*-catenin, 1 : 1000, Cell Signaling Technology, USA; GAPDH, 1 : 2000, Abcam, USA) overnight at 4°C, respectively. After washed with TBST (Beyotime Biotechnology, China), the membranes were incubated with IgG conjugated to horseradish peroxidase (1 : 2000; Santa Cruz Biotechnology, USA). The protein bands were visualized by using an ECL kit (Merck Millipore, USA). The relative expression level of specific protein was quantified by the optical density of the target band against that of GAPDH using a Bio-Rad image analyzer (Bio-Rad, USA).

### 2.11. Statistical Analysis

The results were analyzed through the GraphPad software, and the data are expressed as mean ± standard deviation (SD) of three biological replicates. Student's *t* test was used for comparisons between the two groups, while one-way ANOVA was used for analyzing more than two groups. *p* values <0.05 were considered statistically significant.

## 3. Results

### 3.1. Characterization of SMSCs

**Figure 1:**
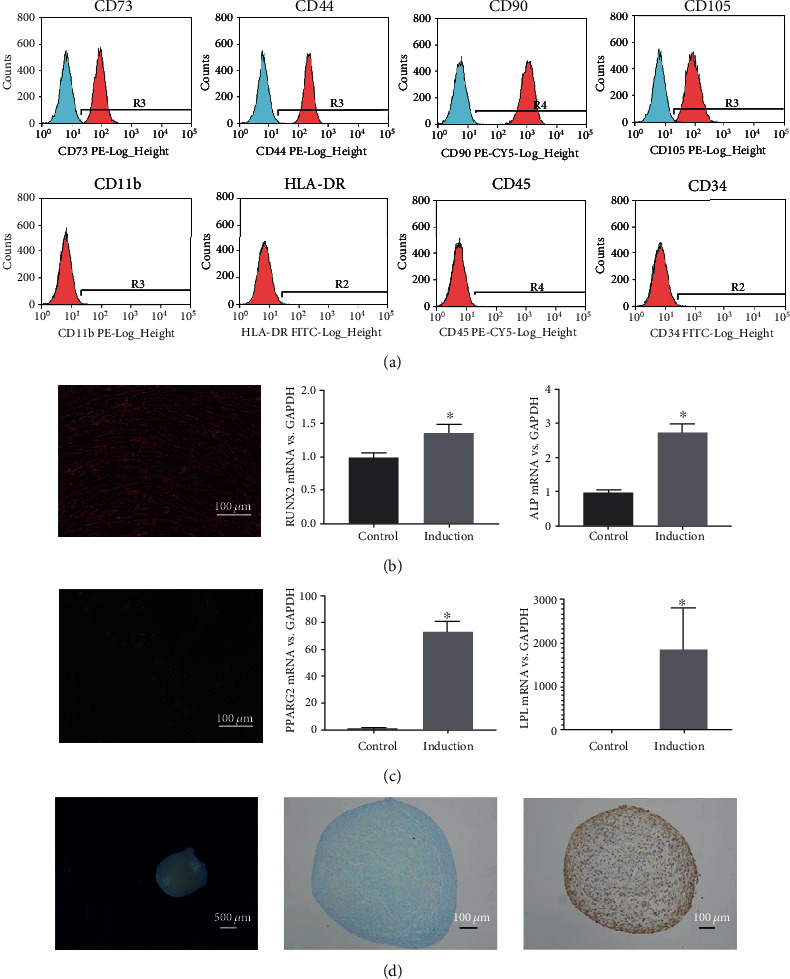
Characterization of SMSCs in the temporomandibular joint. (a) SMSCs tested positive for CD90, CD73, CD105, CD44, and negative for CD34, CD11b, CD45, HLA-DR. (b) Alizarin red staining of SMSCs cultured in osteogenic medium for 2 weeks, and the relative levels of RUNX2 and ALP mRNA in the control and osteogenic induction groups (scale bars = 100 *μ*m; ^∗^ indicates *p* < 0.05). (c) Oil red O staining of SMSCs after adipogenic induction for 2 weeks, and the relative levels of PPARG2 and LPL mRNA in the control and adipogenic induction groups (scale bars = 100 *μ*m; ^∗^ indicates *p* < 0.05). (d) Cartilage pellet formed in the chondrogenic medium over 2 weeks (scale bars = 500 *μ*m); alcian blue staining and collagen II immunohistochemical staining of cells in the pellet (scale bars = 100 *μ*m).

SMSCs grew with adherence and expressed CD73, CD105, CD44, and CD90 but not CD11b, HLA-DR, CD34, and CD45 (Figure 1(a)).

Additionally, SMSCs exhibited multipotent differentiation potential under specific induction. After osteogenic medium induction, the differentiation of SMSCs was evidenced by significant calcium mineralization with alizarin red staining. The gene expression of osteogenic markers, RUNX2 and ALP, in the induced group was higher than that observed in the control group (*p* = 0.013, *p* < 0.001) (Figure 1(b)). In addition, we observed lipid droplets and positive staining for oil red O after 2 weeks of adipogenic differentiation. The gene expression of adipogenic markers, PPARG2 and LPL, in the induced group was higher than that observed in the control group (*p* = 0.028, *p* < 0.001) (Figure 1(c)). In addition, in the chondrogenic differentiation group, the cartilage pellets were observed. The cartilage pellet sections showed positive alcian blue staining, and collagen II was highly expressed in the immunohistochemical analysis (Figure 1(d)).

### 3.2. IL-1*β* Inhibits the Chondrogenesis of SMSCs

**Figure 2:**
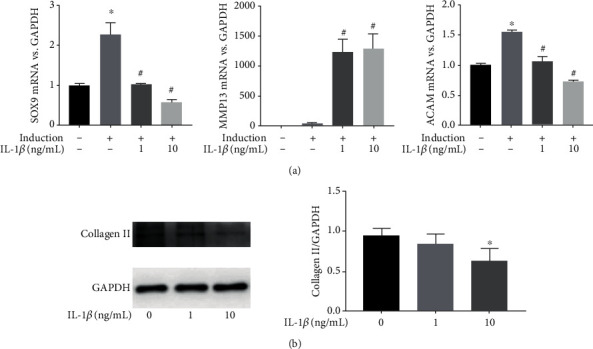
IL-1*β* impedes the chondrogenesis of SMSCs. (a) Relative levels of SOX9, MMP13, and ACAN mRNA during the chondrogenic differentiation of SMSCs treated with or without IL-1*β* for 2 weeks (^∗^ indicates *p* < 0.05 compared with the control group; ^#^ indicates *p* < 0.05 compared with the chondrogenic induction group without IL-1*β* stimulation). (b) Collagen II protein expression was detected by a western blot assay (^∗^ indicates *p* < 0.05 compared with the chondrogenic induction group without IL-1*β* stimulation).

IL-1*β* initiates a cascade of inflammatory responses in TMD and ultimately leads to tissue destruction. We observed that the chondrogenic differentiation ability of SMSCs decreased when cultured in chondrogenic induction medium supplemented with 1 or 10 ng/mL IL-1*β* for 2 weeks. Furthermore, the gene expression of MMP13 increased (*p* < 0.001), while that of SOX9 and ACAN decreased with increasing concentrations of IL-1*β* (*p* < 0.001, *p* < 0.001) (Figure 2(a)). In addition, the western blotting results showed that collagen II expression decreased accordingly in the IL-1*β*-mediated chondrogenic induction group (*p* = 0.049) (Figure 2(b)).

### 3.3. IL-1*β* Inhibits the Autophagy of SMSCs during Chondrogenic Induction

**Figure 3:**
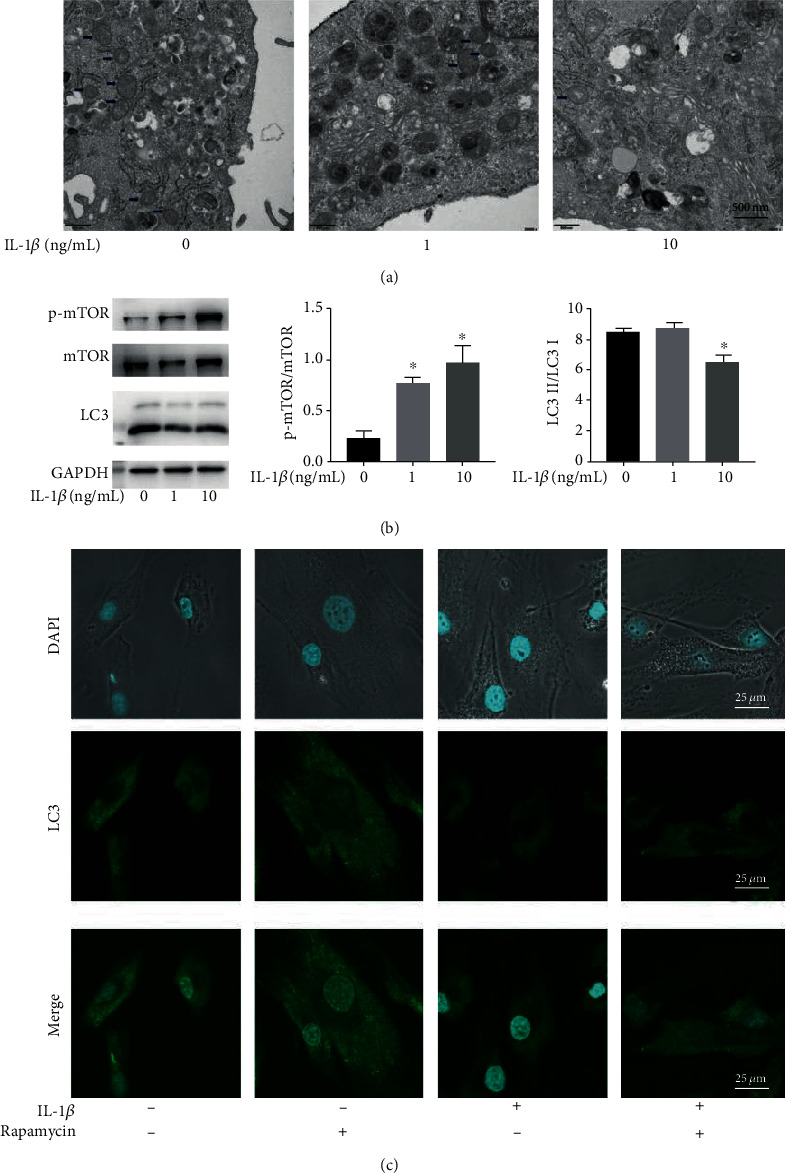
IL-1*β* inhibits autophagy in SMSCs by activating mTOR. (a) Transmission electron microscopy images showing autophagosomes in SMSCs after chondrogenic induction for 24 h (arrows, scale bars = 500 nm). (b) Expression of the autophagy-related proteins mTOR and LC3 in SMSCs treated with IL-1*β* (^∗^ indicates *p* < 0.05 compared with the control group). (c) SMSCs were seeded on glass coverslips for 24 h in the chondrogenic induction culture medium, chondrogenic induction culture medium supplemented with 100 nM rapamycin, chondrogenic induction culture medium supplemented with 10 ng/mL IL-1*β*, or with a 1-h preincubation with 100 nM rapamycin prior to the addition of 10 ng/mL IL-1*β* in the chondrogenic induction culture medium, and LC3-positive punctures were observed in an immunofluorescence assay (scale bars = 25 *μ*m).

To investigate the role of autophagy in the chondrogenesis of inflammatory SMSCs, we assessed the number of autophagosomes and the expression of the autophagy-related indicators mTOR and LC3 in the different groups. Transmission electron microscopy images showed that the number of autophagosomes was decreased in the high IL-1*β* stimulation group compared with that observed in the control group 24 h after chondrogenic induction (Figure 3(a)). Furthermore, the protein expression of p-mTOR was upregulated (*p* = 0.018), while the ratio of LC3II/LC3I was decreased (*p* = 0.038) in SMSCs after a 24 h treatment with IL-1*β* based on western blot results (Figure 3(b)). Moreover, immunofluorescence assay results showed that the number of LC3-positive punctures in SMSCs significantly decreased 24 hours after treatment with IL-1*β* to mediate chondrogenic induction (Figure 3(c)). When the cells were treated with rapamycin, autophagy was activated, and the number of LC3-positive punctures in SMSCs was increased accordingly (Figure 3(c)).

### 3.4. Rapamycin Promotes the Migration and Chondrogenesis of SMSCs

**Figure 4:**
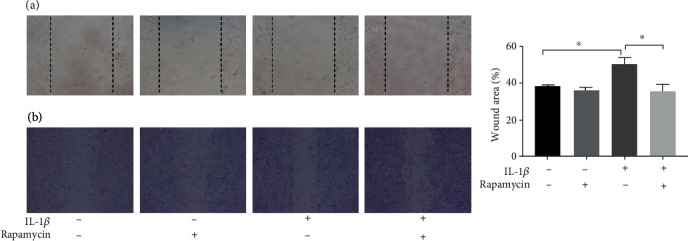
Rapamycin promotes the migration of SMSCs. (a) Scratch was made in SMSCs, which were seeded in a 24-well plate. (b) The scratch width was stained with crystal violet solution in each group after 24 h (^∗^ indicates *p* < 0.05).

MSC migration to damaged tissue and colonization are prerequisites for tissue repair. The crystal violet staining results showed that the scratch width was larger at 24 hours in the IL-1*β* treatment group than that observed in the control group (*p* = 0.002). In the rapamycin group, scratch closure was significantly increased compared to that observed in SMSCs without rapamycin (*p* < 0.001) (Figure 4), suggesting that rapamycin-induced autophagy enhanced the migration of SMSCs.

**Figure 5:**
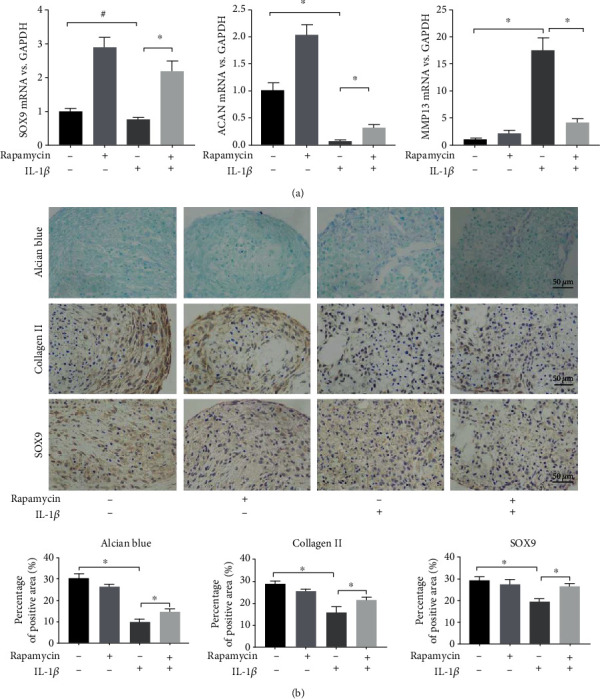
Rapamycin enhances the chondrogenesis of SMSCs. (a) Relative levels of SOX9, ACAN, and MMP13 mRNA in each group in response to chondrogenic induction for 2 weeks as detected by qRT-PCR (^∗^ indicates *p* < 0.05, ^#^ indicates *p* < 0.10). (b) Alcian blue staining and collagen II- and SOX9-positive immunostained cells in the cartilage pellet of each group (scale bars = 50 *μ*m) (^∗^ indicates *p* < 0.05).

With respect to chondrogenesis, rapamycin significantly increased the gene expression of SOX9 and ACAN in cartilage pellets in the presence of IL-1*β* (*p* < 0.001, *p* = 0.004) but decreased the MMP13 expression (*p* < 0.001) (Figure 5(a)). Similar results were observed by histological staining with respect to the formation of alcian blue deposits, and the number of SOX9- and collagen II-positive cells was increased in cartilage pellets pretreated with rapamycin compared to that observed in the group treated with IL-1*β* alone (*p* = 0.040, *p* = 0.004, *p* = 0.010) (Figure 5(b)).

### 3.5. Rapamycin Upregulates the Autophagy of SMSCs through GSK3*β*

**Figure 6:**
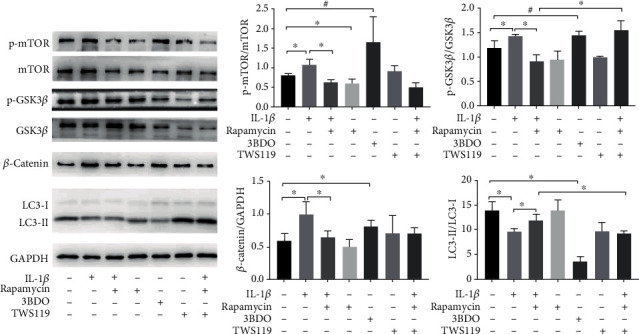
Rapamycin upregulates autophagy in SMSCs through GSK3*β*. Protein expression levels of mTOR, GSK3*β*, *β*-catenin, and LC3 in SMSCs in each group were detected by western blot assays (^∗^ indicates *p* < 0.05, ^#^ indicates *p* < 0.10).

The inhibition of the GSK3*β* activity and the expression of *β*-catenin can activate the Wnt/*β*-catenin signalling pathway, which was reported to affect the cartilage function and metabolism. To study the effect of GSK3*β* inhibition in autophagy in chondrogenesis, we examined the changes of GSK3*β*, *β*-catenin, and LC3 expression in inflammatory SMSCs by western blot assays. We observed that cells treated with IL-1*β* had increased levels of p-GSK3*β*ser9 and *β*-catenin (*p* = 0.047, *p* = 0.035). Consistent with the effect of IL-1*β*, cells treated with 3BDO (50 *μ*g/mL), an activator of mTOR signalling, led to autophagy inhibition and higher levels of p-GSK3*β*ser9 and *β*-catenin than was observed in the control treatment (*p* = 0.059, *p* = 0.048). In contrast, rapamycin pretreatment decreased the p-GSK3*β*ser9 and *β*-catenin in the SMSCs produced by IL-1*β* treatment (*p* = 0.002, *p* = 0.0497). The activity of GSK3*β* is inhibited when phosphorylated at serine 9. Furthermore, the addition of the GSK3*β* inhibitor TWS119 in the IL-1*β*+rapamycin treatment group disrupted the autophagic activity and reduced the expression of LC3-II/LC3-I (*p* = 0.026) (Figure 6). These results show that GSK3*β* may be a key downstream target of mTOR and play a role in regulating autophagy.

## 4. Discussion

The prevalence of TMD is increasing and has become the second most important musculoskeletal disorder affecting the quality of life [12]. Along with inflammatory progression, the injured articular cartilage in TMD has limited capacity to heal. The application of MSCs and tissue engineering technology to repair cartilage destruction has become a hotspot in the field of TMD treatment[13]. MSCs can be derived from specific tissues including the bone marrow, blood, teeth, muscle, and skin, showing different advantages in the proliferation efficiency or pluripotency capacity. SMSCs have been shown to be the best choice for cartilage repair [1, 14]. A study showed the number of synovial fluid-derived MSCs, which is similar to SMSCs, increased along with the radiological grading of osteoarthritis [15]. However, the cartilage repair ability of the existing MSCs still needs to be further elucidated.

Recent studies have revealed that the local microenvironment can affect the biological potential of stem cells by initiating signal transduction. For example, inflammatory factors or bacterial components in periodontitis can affect the osteogenic differentiation of periodontal ligament MSCs [16, 17]. In response to IL-1*β*, TNF-*α*, or coculture with leukocytes, the osteogenic differentiation ability of adipose MSCs is enhanced, while the chondrogenic and adipogenic differentiation ability is impaired[18, 19]. Our previous study also showed that in the inflammatory microenvironment, the synovial fluid-derived MSCs exhibit an increased secretion of IL-6 and IL-8, which are the primary catabolic cytokines of the cartilage matrix [20]. Furthermore, SMSCs from the hip joints of individuals with osteoarthritis showed greater osteogenic and adipogenic potentials, whereas the cells from patients with femoroacetabular impingement syndrome showed greater chondrogenic potential [2]. Thus, the properties of MSCs are closely associated with specific pathologies, and inflammatory cytokines are one of the main obstacles affecting the multilineage differentiation. During the process of chondrogenesis, ACAN and collagen II are the main cartilage matrix components. SOX9 is one of the primary regulators of chondrogenic differentiation and promotes the expression of chondrocyte matrix components (including ACAN and collagen II). By contrast, MMP13 is a major matrix proteinase that targets collagen II, which plays an important role in cartilage degradation. Therefore, we detected the relative expression of the above related biomarkers in the presence of IL-1*β*, which is the main inflammatory cytokine in TMD [20], and found that the chondrogenesis of SMSCs decreased.

Autophagy dynamically maintains cell self-renewal and metabolism. Autophagy activation has been shown to occur during the multidifferentiation of MSCs, including the osteogenic differentiation of the dental pulp MSCs [21], the adipogenic [22] and neuron-induced differentiation [23] of bone marrow MSCs, and the skeletal muscle differentiation of human tonsil-derived MSCs [24]. However, the role of autophagy in chondrogenesis is seldom reported in the literature. Moreover, decreased autophagy results in the accumulation of reactive oxygen species, which leads to mitochondrial dysfunction and promotes the apoptosis of synovial cells [4]. In contrast, the decreased levels of autophagy have been observed in the synovium of adjuvant arthritis rats, leading to the excessive proliferation of synovial cells [5]. The dual roles of autophagy in the regulation of synovial cells are not conducive to the homeostasis of the intra-articular environment. Therefore, we speculated that autophagy may be involved in the disabling of chondrogenic differentiation by SMSCs, and the results of this study demonstrated that the autophagy levels decreased in chondrogenic induction experiments with IL-1*β*. However, the relationship between autophagy and the chondrogenic differentiation of SMSCs needs to be explored in further experiments. Decreased autophagy-related gene expression has been observed in damaged chondrocytes, and enhanced autophagy can slow the pathological progression of osteoarthritis [25, 26]. In addition, autophagy deficiency may affect the growth plate chondrocytes, leading to cartilage development delays [27, 28]. Autophagy has been suggested to be involved in the development and metabolism of cartilage. However, the effect of autophagy on cellular processes may vary depending on the cell sources with specific activating signals. For example, Wang et al. reported that low-intensity pulsed ultrasound promotes rat bone marrow MSC chondrogenesis by inhibiting beclin1-mediated autophagy[29]. In addition, Zhang et al. observed that low-intensity pulsed ultrasound increased the autophagy level of macrophages through SQSTM1-dependent autophagic degradation in osteoarthritis [30]. Whether autophagy plays a role in the chondrogenic differentiation of SMSCs and the associated mechanism has not yet been reported. mTOR is a conserved protein kinase and the key regulator of autophagy. mTOR inhibition-mediated autophagy is an important signalling pathway in the differentiation of MSCs [21]. We found that the expression of mTOR was increased during the IL-1*β*-induced chondrogenesis. Further studies showed that rapamycin increased the autophagy level and promoted the migration and chondrogenic differentiation of SMSCs in response to IL-1*β*. These results suggest that rapamycin-activated autophagy can promote the chondrogenic differentiation of SMSCs in the inflammatory microenvironment. This finding may explain the protective role of rapamycin in articular cartilage from osteoarthritis [31, 32]. Intriguingly, it was demonstrated that the activity of mTOR is different in the early and late stages of osteogenic differentiation [21]. Therefore, the role of autophagy throughout the process of chondrogenic differentiation should be elucidated in greater detail in future studies.

As a serine protein kinase, GSK3*β* is widely involved in cell growth, differentiation, and signal transduction. Furthermore, GSK3*β* was shown to promote the chondrogenic differentiation of MSCs and maintain the phenotype of chondrocytes in vitro by stimulating the expression of collagen II and aggrecan [33]. Additionally, the activation of GSK3*β* participates in the ubiquitination of *β*-catenin and the degradation of the proteasome. *β*-catenin, a key protein of Wnt signaling, also affects the differentiation and maturation of chondrocytes and promotes the degradation of the cartilage matrix [34]. Consistent with these findings, our results demonstrated that the activity of GSK3*β* was inhibited and the *β*-catenin expression was increased in SMSCs in response to IL-1*β*, having an unfavourable effect on chondrogenesis. The regulation of GSK3*β* can have a dual role in many physiological processes. Some studies have demonstrated that GSK3*β* inhibition promotes autophagy by targeting the mTOR pathway or lysosomal biogenesis [35, 36]. However, p-GSK3*β*ser9 can function as a downstream regulator of mTOR [37]. GSK3*β* inhibition via the high expression of p-GSK3*β*ser9 has been shown to cause resistance to rapamycin in cancer cell lines [38, 39]. Furthermore, GSK3*β* inhibition is known to lead to the accumulation of *β*-catenin, which has been shown to inhibit autophagy [40]. Different outcomes suggest that the role of GSK3*β* in autophagy depends on cell lines, culture conditions, and duration of exposure. Our results showed that the expression of p-GSK3*β*ser9 and *β*-catenin is increased and that autophagy is inhibited in SMSCs following mTOR activator. Furthermore, a GSK3*β* inhibitor reversed the effect of rapamycin-induced autophagy in inflammatory SMSCs. Thus, GSK3*β* may be a key downstream target of mTOR that affects the level of autophagy. However, the effect of this specific relationship on chondrogenesis still requires additional verification. In our study, we investigated the role of autophagy in the chondrogenesis of SMSCs in response to IL-1*β* and found that rapamycin-induced autophagy promotes the chondrogenic differentiation of SMSCs through GSK3*β* and *β*-catenin.

There are several limitations to this study. The SMSCs used in this study were obtained from patients without distinguishing the differences in age and sex, knowing these variables are important since they can lead to differences in autophagy and chondrogenic differentiation observed in vitro. Furthermore, the long-term and stable effects of rapamycin in inducing autophagy should be conducted through in vivo experiments in future studies.

## 5. Conclusions

The inhibition of autophagy in SMSCs in response to IL-1*β* may be an important factor and mechanism for insufficient cartilage repair. This study showed that rapamycin can induce autophagy through GSK3*β* and promote the chondrogenic differentiation of SMSCs. These findings may shed light on the development of new therapeutic strategies for TMD.

## Figures and Tables

**Table 1:** Oligonucleotide primers used in PCR.

Gene	Primer sequence
*RUNX2*	Fw 5′-TCAACGATCTGAGATTTGTGGG-3′
	Rv 5′-GGGGAGGATTTGTGAAGACGG-3′
*ALP*	Fw 5′-CAGTTGAGGAGGAGAACCCG-3′
	Rv 5′-CACATATGGGAAGCGGTCCA-3′
*PPARG2*	Fw 5′-GCAAACCCCTATTCCATGCTG-3′
	Rv 5′-CACGGAGCTGATCCCAAAGT-3′
*LPL*	Fw 5′-CAAGAGTGAGTGAACAAC-3′
	Rv 5′-AATTATGCTGAAGGACAAC-3′
*SOX9*	Fw 5′-ACACACAGCTCACTCGACCTTG-3′
	Rv 5′-AGGGAATTCTGGTTGGTCCTCT-3′
*MMP13*	Fw 5′-GACTGGTAATGGCATCAAGGGA-3′
	Rv 5′-CACCGGCAAAAGCCACTTTA-3′
*ACAN*	Fw 5′-CTTCCGCTGGTCAGATGGAC-3′
	Rv 5′-CGTTTGTAGGTGGTGGCTGTG-3′
*GAPDH*	Fw 5′-GACAGTCAGCCGCATCTTCT-3′
	Rv 5′-TTAAAAGCAGCCCTGGTGAC-3′

## Data Availability

The data used to support the findings of this study are included in the article.
